# C286, an orally available retinoic acid receptor β agonist drug, regulates multiple pathways to achieve spinal cord injury repair

**DOI:** 10.3389/fnmol.2024.1411384

**Published:** 2024-08-20

**Authors:** Maria B. Goncalves, Yue Wu, Earl Clarke, John Grist, Julien Moehlin, Marco Antonio Mendoza-Parra, Carl Hobbs, Barret Kalindjian, Henry Fok, Adrian P. Mander, Hana Hassanin, Daryl Bendel, Jörg Täubel, Tim Mant, Thomas Carlstedt, Julian Jack, Jonathan P. T. Corcoran

**Affiliations:** ^1^Neuroscience Drug Discovery Unit, Wolfson Sensory, Pain and Regeneration Centre, King's College London, Guy's Campus, London, United Kingdom; ^2^UMR 8030 Génomique Métabolique, Genoscope, Institut François Jacob, CEA, CNRS, University of Évry-val-d'Essonne, University Paris-Saclay, Évry, France; ^3^NIHR Biomedical Research Centre at Guy's and St Thomas' NHS Foundation Trust and King's College London, London, United Kingdom; ^4^Centre for Trials Research, Cardiff University, Cardiff, United Kingdom; ^5^Surrey Clinical Research Centre, University of Surrey, Guildford, United Kingdom; ^6^Richmond Pharmacology Limited, London, United Kingdom

**Keywords:** retinoic acid, drug, pathway analysis, nerve regeneration and repair, human data

## Abstract

Retinoic acid receptor β2 (RARβ2) is an emerging therapeutic target for spinal cord injuries (SCIs) with a unique multimodal regenerative effect. We have developed a first-in-class RARβ agonist drug, C286, that modulates neuron-glial pathways to induce functional recovery in a rodent model of sensory root avulsion. Here, using genome-wide and pathway enrichment analysis of avulsed rats' spinal cords, we show that C286 also influences the extracellular milieu (ECM). Protein expression studies showed that C286 upregulates tenascin-C, integrin-α9, and osteopontin in the injured cord. Similarly, C286 remodulates these ECM molecules, hampers inflammation and prevents tissue loss in a rodent model of spinal cord contusion C286. We further demonstrate C286's efficacy in human iPSC-derived neurons, with treatment resulting in a significant increase in neurite outgrowth. Additionally, we identify a putative efficacy biomarker, S100B, which plasma levels correlated with axonal regeneration in nerve-injured rats. We also found that other clinically available retinoids, that are not RARβ specific agonists, did not lead to functional recovery in avulsed rats, demonstrating the requirement for RARβ specific pathways in regeneration. In a Phase 1 trial, the single ascending dose (SAD) cohorts showed increases in expression of RARβ2 in white blood cells correlative to increased doses and at the highest dose administered, the pharmacokinetics were similar to the rat proof of concept (POC) studies. Collectively, our data suggests that C286 signalling in neurite/axonal outgrowth is conserved between species and across nerve injuries. This warrants further clinical testing of C286 to ascertain POC in a broad spectrum of neurodegenerative conditions.

## Introduction

Spinal cord injuries (SCIs) are a complex clinical condition that requires multitarget therapies involving a combination of promoting axonal regrowth and overcoming the blocks associated with glial scarring (Afshari et al., 2009) to attain significant functional recovery. A plethora of preclinical studies show that retinoic acid (RA) signalling can induce axonal regeneration through specific activation of the retinoic acid receptor (RAR) β in the injured neurons. RARβ signalling is involved in the regeneration of axons in a variety of central nervous system (CNS) injuries, including optic nerve (Koriyama et al., 2013), corticospinal tract (Yip et al., 2006), and avulsion (Goncalves et al., 2015, 2019c). The clinical exploitation of RARβ as a therapeutic target has been curtailed by the absence of RARβ agonists with good drug-like properties. We have recently developed C286, an oral RARβ agonist drug that has been tested in a Phase 1 trial and demonstrated favourable safety and pharmacokinetic profiles at the envisaged desired pharmacological exposure ranges.

Here we used messenger ribonucleic acid (mRNA) sequencing and analysis of differentially regulated pathways to identify a regenerative pathway signature of C286 in the injured spinal cord (SC), corroborating a multimodal effect. Amongst the top differentially regulated pathways were the extracellular and transmembrane domains. Through protein expression analysis, we found that C286 upregulates tenascin-C (TN-C), integrin α9 and osteopontin (OPN) in the lesioned SC. Aside from modulating multiple regenerative transcription patterns, we further demonstrate the potential wider clinical applicability of C286 by demonstrating its efficacy in restoring locomotor function in a spinal contused model. The agonist significantly reduced inflammation and tissue loss in the injured cord, promoted chondroitin sulphate proteoglycan (CSPG) clearance via decorin, and remodelled extracellular matrices (ECMs) and adhesion molecules, mechanisms also seen in the avulsed rat (Goncalves et al., 2019c). We demonstrate target engagement at the lesion site in nerve-injured rats and report that in healthy human participants (Trial ID: https://www.isrctn.com/ISRCTN12424734; registration date: 18 July 2018). C286 engages its target receptor in white blood cells (WBC) at oral doses corresponding to systemic exposures associated with functional recovery in the rat. In avulsed rats, plasma S100B was significantly higher from day 21 to day 41 post-injury in agonist-treated rats compared to vehicle-treated rats. Finally, to show that specific activation of the RARβ was necessary to elicit regeneration, we used five clinically approved retinoids that are not RARβ selective and show they do not induce recovery in the avulsed rat model. Our data demonstrates that C286, a first-in-class drug, has a multifactorial action on nerve injury that warrants its use in proof of concept (POC) trials for a variety of SCIs.

## Materials and methods

### Potency/selectivity of the compound

#### Transient cell transfections for transactivation studies

The vectors used have been previously described (Goncalves et al., 2019a). The day before transfection, cos-7 cells are seeded on 24-well culture plates at a density of 5 × 10^4^ cells per well so that they are 50%−70% confluent on the day of transfection. Cells were cotransfected with 0.1 μg of reporter vector pRARE-tk-Luc that allows the expression of the reporter gene firefly luciferase, 0.05 μg of the given pSG5-RAR expression vector or given human RAR LBD expression vector (Invitrogen), and 0.02 μg of the pRLnull vector that encodes the Renilla luciferase (Promega) as an internal control to normalise for variations in transfection efficiency. The total DNA (0.17 μg/well) was diluted with 25 μl of Dulbecco's Modified Eagle Medium (DMEM) mixed with 2 μl of Plus reagent and LipofectAMINE (0.6 μl) with 25 μl of DMEM are added and incubated for 15 min at room temperature. The cells were washed twice with DMEM to eliminate serum traces, and 200 μl of serum-free DMEM was added per well. The mixture was added to the cells and incubated at 37°C. After 3 h of incubation, the culture volume was increased to 1 ml per well with DMEM-supplemented 10% serum (Invitrogen). Twenty-four hours after transfection, the cells were washed and then treated with the appropriate retinoid in a serum-free medium for 12 h. The cells were then washed twice with cold phosphate-buffered saline (PBS), lysed with 200 μl per well of passive lysis buffer (Promega), and incubated for 15 min at room temperature.

### Dual luciferase assays

Firefly luciferase and Renilla luciferase activities were determined on 10 μl of lysate using the Dual Luciferase Assay System Kit (Promega). The light intensity was determined with a luminometer after injecting 50 μl of LAR II reagent (firefly luciferase substrate) and 50 μl of Stop and Glo reagent (Renilla luciferase substrate) successively. All the experiments were carried out in triplicate, and the firefly luciferase activity is normalised with the Renilla activity (efficiency of transfection) and with the protein content.

### RNA extraction and sequencing

Total RNA isolation and Reverse transcription-quantitative polymerase chain reaction (RT-qPCR) analysis.

Frozen micro-dissected SC samples were lysed with Teflon-glass homogenisers and QIA shredder columns (Qiagen). Total RNA was isolated with the Qiagen RNeasy^®^ Mini kit. The RNA was assessed for purity and quantity using the Nanodrop 1000 spectrophotometer and assessed for quality on the Agilent Bioanalyzer 2100. RNA was reverse transcribed with a QuantiTect^®^ Reverse Transcription Kit (Qiagen).

### mRNA library preparation

**Figure 1:**
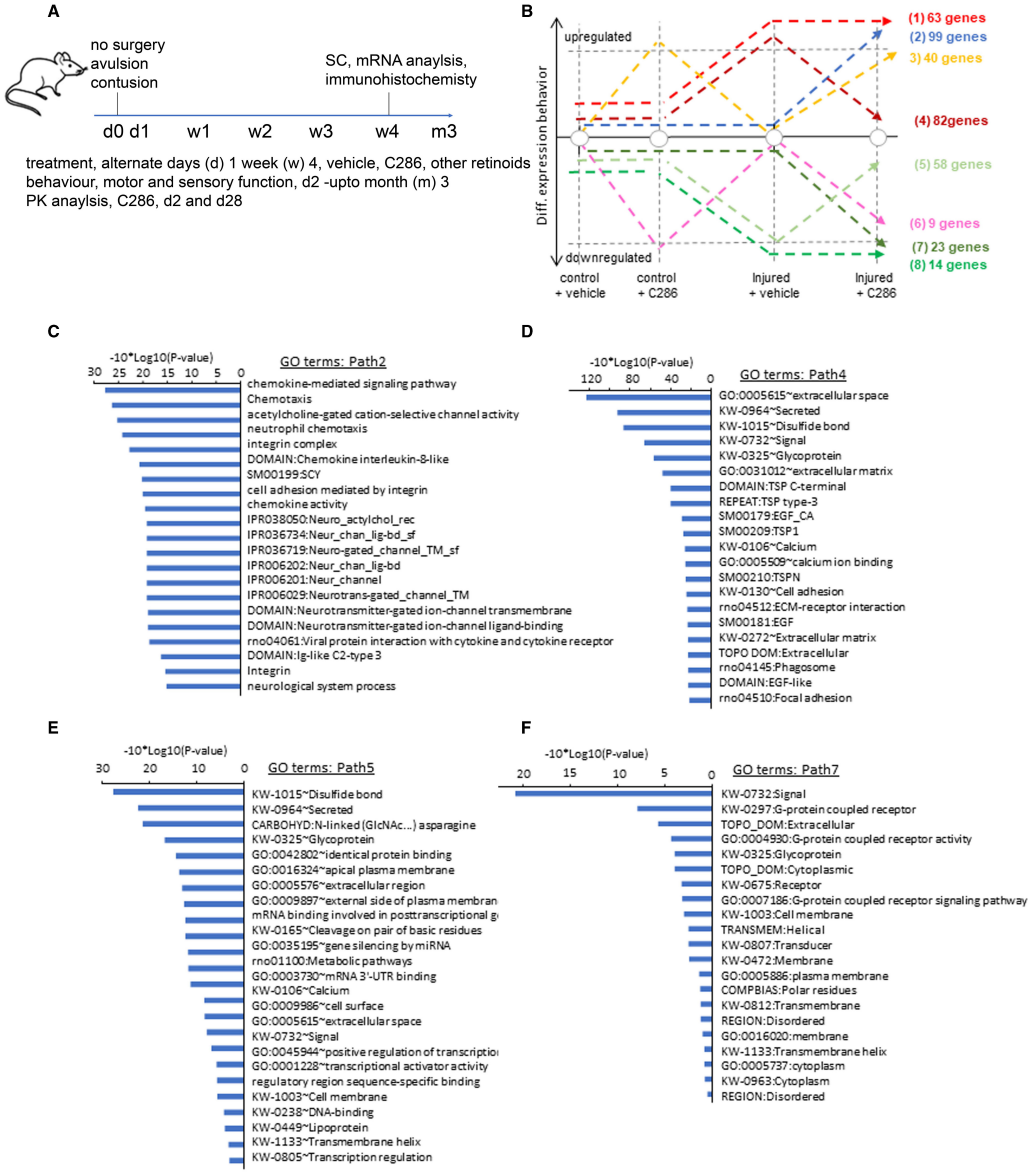
C286 modulates multiple pathways in the injured SC. **(A)** Schematic of experimental procedures; **(B)** number of genes regulated by the drug in various treatments; (**C–F**) Gene Ontology (GO) analysis performed per co-expression path, GO enrichment confidence [−10*log10 (*p*-value)]; **(C, E)** GO terms for paths differentially upregulated in injured SC by C286 compared to vehicle-treated; and **(D, E)** GO terms for paths differentially downregulated in injured SC by C286 compared to vehicle-treated.

Four hundred ng of intact, high-quality total RNA (RIN>7.9) from each sample was then used as input to generate libraries for RNA sequencing using the New England Biolabs (NEB) Next Ultra II Directional Kit (NEB, Cat. No. E7760S) following the manufacturer's recommendations. This protocol involved an initial step of mRNA selection using a poly-A isolation module (NEB, Cat. No. E7490) to select for mRNA with a mature poly-A tail, followed by fragmentation before first cDNA synthesis and barcoding second-strand cDNA synthesised with indices for Illumina sequencing for final library amplification (10 cycles). The resulting libraries (342–421 bp) were assessed on the Bioanalyzer 2100 for purity. The NEBNext Library Quant Kit for Illumina (NEB, Cat. No. E7630L) was used to calculate the quantity of each library. The quantification data was used to pool the libraries in equal molarity before performing a quality control (QC) run on the MiSeq [MiSeq Reagent Kit v3 (150-cycle); Cat. No. MS-102-3001]. Furthermore deep sequencing was performed on the pooled library over two lanes using a HiSeq4000 (by GENEWIZ) to generate roughly 26 million reads per sample. RNA-Seq Data Analysis Samples were aligned to the reference genome (Rattus_norvegicus Rnor_6.0.94) with Bowtie2 (Langmead and Salzberg, 2012). Aligned sequenced reads per sample were associated with annotated genes with HTSeq (https://htseq.readthedocs.io/en/release_0.11.1/count.html), followed by an inter-sample quantile normalisation to correct for technical differences. Differential expression analysis (relative to the samples issued from non-injured and vehicle-treated animals) has been performed with Deseq2 (Love et al., 2014). Differentially expressed genes assessed in various conditions (Non-injured +C286; Injured +vehicle; Injured +C286) were stratified over three major states (induced, repressed or non-differentially expressed) such that a total of 26 hypothetical combinatorial co-expression events were inferred. Among them, only eight appeared as biologically significant situations where the effect of the ligand C286 could be inferred (Figure 1G). To support this hypothesis, all combinatorial co-expression events were analysed for Gene Ontology (GO) enrichment (DAVID Bioinformatics Resources, Frederick, MD, USA) (da Huang et al., 2009), demonstrating relevant enrichment GO terms preferentially in the case of the selected biologically selected co-expression paths.

### Cell culture

Human iPSC-derived cerebral cortical neurons (HyCCNs; Ax0026) were cultured and differentiated as per the manufacturer's guidelines (www.axolbio.com/page/neural-stem-cells-cerebral-cortex). Cells were plated at 30K per cm^2^, and treatments were performed with either vehicle or 0.1 μM C286 for 3 days on three independent cultures.

### Immunochemistry quantification

Quantification of protein levels by immunofluorescence (IF) was done as previously described (Herrmann et al., 2010). In brief, positively stained areas were quantified as the pixels of immunoreactivity above a threshold level per unit area using the Zeiss Zen Blue Edition software. The threshold value was set to include a fluorescent positive signal and exclude background staining. The threshold values for a given section and stain remained the same throughout the study, and the quantifications were done by an operator blinded to the treatments. Confocal microscopy multichannel fluorescence (DAPI–FITC–Texas Red filter set) images were captured using a Zeiss LSM 700 laser-scanning confocal microscope. For high-magnification images, a 63× oil-immersion Aprochromat objective (Carl Zeiss) was used. Settings for gain, aperture, contrast, and brightness were optimised initially and held constant throughout each study so that all sections were digitised under the same conditions of illumination. Channels were imaged sequentially to eliminate bleed-through, and multichannel image overlays were obtained using Adobe Photoshop 7.0 (Adobe Systems).

### Surgery and drug treatments

All procedures followed the UK Home Office guidelines and the Animals (Scientific Procedures) Act of 1986. Male Sprague–Dawley rats, weighing 220–250 grammes were used throughout the study. All animal care and experimental procedures complied with the Animals (Scientific Procedures) Act, 1986 of the UK Parliament, Directive 2010/63/EU of the European Parliament, and the Guide for the Care and Use of Laboratory Animals published by the US National Institutes of Health (NIH Publication No. 85–23, revised 1996). Animal studies are reported in compliance with the ARRIVE (Animal Research: Reporting of *In Vivo* Experiments) guidelines (Kilkenny et al., 2010; McGrath and Lilley, 2015). All surgery, behavioural testing, and analyses were performed using a randomised block design and in a blinded fashion. Allocation concealment was performed by having the treatment stocks coded by a person independent of the study. Codes were only broken after the end of the study. Animals were housed in groups of three to four in Plexiglas cages with tunnels and bedding on a 12:12 h light–dark cycle and had access to food and water *ad libitum*.

Rats were anaesthetised using a mixture of ketamine (60 mg/kg) and medetomidine (0.25 mg/kg) administered intraperitoneally. During the surgery, the rats were placed on a controlled heating pad to maintain a temperature of 37 ± 1°C. All the rats survived the surgery.

After the surgery, the rats were hydrated with physiological saline (2 ml, s.c.). Anaesthesia was reversed with an intramuscular (IM) injection of 0.05 ml (1 mg/kg) atipamezole hydrochloride (Antisedan^®^ Pfizer Animal Health, Exton, PA). Animals were kept in a heated recovery box until fully conscious, and analgesia (buprenorphine, 0.01 mg/kg, subcutaneously) was given after suturing and recovery.

Sensory root avulsion model: in male Sprague–Dawley rats (eight per treatment group for each set of experiments), C5–C8 and T1 dorsal roots were cut flush with the SC surface. The cut ends of the dorsal roots were subsequently introduced through slits in the pia mater and positioned superficially in the SC adjacent to where they had been cut, as previously described (Goncalves et al., 2015).

Spinal cord contusion model: in male Sprague–Dawley rats (four per treatment group for each set of experiments), following a laminectomy at T10, the vertebral column was stabilised, and the impactor probe was positioned above the SC. An impact force of 150 kdyn was delivered to the exposed SC through the intact dura, as previously described (James et al., 2011).

Rats were treated with vehicle or a specific RARβ agonist, C286 (synthesised by Sygnature Chemical Services, Nottingham, UK), given by oral gavage (po) three times a week for 4 weeks at 3 mg/kg. For comparison of the biological effects of the different retinoids, rats were treated with C286, Am80 (Tamibarotene), atRA (Tretinoin), 9-cis-RA, Acitretin, and 13-cis-RA (Isotretinoin), all purchased from Sigma Aldrich, UK, by intraperitoneal injections (i.p.) at 1 mg/kg three times a week for 4 weeks.

Animals were culled after 4, 5, or 6 weeks of treatment, as indicated. Rats were perfused transcardially with a heparinised 0.9% NaCl solution and 4% paraformaldehyde in 0.1 M phosphate buffer (PB). For the avulsion studies, the spinal cords comprising the lesioned areas were dissected, rapidly removed and post-fixed with 4% paraformaldehyde (in 0.1 M PB) for at least 2 days at room temperature. The tissue was then embedded in paraffin wax, and 5 μm transverse sections were cut throughout each block. Sets of consecutive sections, comprising the lesioned area, were used for immunostaining.

For the spinal contusion studies, after perfusion, a section of the SC was removed (~10mm) with the lesion epicentre located centrally; this was then post-fixed in 4% PFA in 0.1 M PB for 2 h at 4°C before being cryoprotected in 20% sucrose in 0.1 M PB for 48 h. The tissue was then embedded in Optimal cutting temperature compound (OCT) and frozen before being cut into serial 20 μm-thick transverse sections using a cryostat. Sections were mounted on a series of 10 positively charged slides, such that the adjacent sections mounted on each slide represented regions spaced 200 μm apart.

### Quantitative analysis of proteins in the rat tissue

#### Avulsion model

Quantification of osteopontin, integrin α9 and tenascin-C was done as previously described (Goncalves et al., 2015). In brief, positively stained areas were quantified as the pixels of immunoreactivity above a threshold level per unit area. The threshold value was set to include a fluorescent positive signal and exclude background staining. The threshold values for a given section and stain remained the same throughout the study. The number of pixels was measured in a 200 μm^2^ area comprising the Dorsal root entry zone (DREZ) of the implanted severed dorsal roots and the contiguous SC. At least six sections per rat (*n* = 4 per treatment group) were used for these quantifications.

#### SC contusion model

The cavity size for the SC contusion was done as previously described (Tan et al., 2011). Quantification of OX42, NeuN, GFAP, Iba1, CSPG, decorin and tenascin-C intensities was performed in serial transverse SC sections (0–1 mm from the epicentre of the lesion) obtained from four animals per group, comprising a 200 μm^2^ area around the lesion epicentre. Using Axiovision software, the total pixel values for each antibody immunofluorescence staining were measured for each section (six sections per animal). All anatomical quantification was performed by an experimenter blinded to the treatment group.

### Behaviour analysis

The behavioural tests were carried out by experimenters blinded to the treatment for 2 weeks before and for 1–3 months after injury (avulsion model) or 6 weeks post-injury (spinal contusion model). In the tape removal test (Bradbury et al., 2002), adhesive tape (1.5 cm^−1^ cm) was placed on each forepaw separately, and we scored the time taken to sense the tape (indicated by paw shake) and remove the tape (cut-off time point, 1 min). For locomotor tasks (Bradbury et al., 2002), rats were trained to cross a horizontal ladder (18–100 cm with each rung 5 cm apart), and the number of forelimb foot slips (off the beam or below the plane of the ladder) was recorded. Confirmation of injury was obtained by the poor performance of all animals in tape removal and locomotor tasks the day after injury. The behavioural responses of the treated and control groups were compared using a one-way ANOVA followed by Tukey's *post-hoc* test or Fisher test.

### Immunocytochemistry/ immunohistochemistry and antibodies

Immunocytochemistry was carried out as previously described (Goncalves et al., 2005). For paraffin wax (Pwax) embedded SC tissue, sections were first dewaxed in xylene and 100% Industrial Methylated Spirit (IMS), then heated in citric acid (10 mM, pH = 6), until boiling, then washed under a running tap for 5 min. Pwax and frozen sections were washed three times for 5 min each in PBS before incubation with the primary antibody in PBS-0.02% Tween at 4°C overnight. The primary antibody was removed by washing three times for 5 min each in PBS. They were incubated in the secondary antibody for 1 h at room temperature (RT) in PBS-0.02% Tween and then washed in PBS three times for 5 min. Antibodies used were: mouse monoclonal anti-βIII tubulin (Promega, 1:1,000 for immunohistochemistry), chicken polyclonal anti-GFAP (Abcam, 1:300); rabbit polyclonal anti-RARβ (Santa Cruz Biotechnology, Inc, 1:100 for immunohistochemistry), goat polyclonal anti-CGRP (Abcam, 1:200); mouse monoclonal anti-GAP-43 (Millipore, 1:20); mouse monoclonal anti-NF160 (Sigma Aldrich, 1:200); mouse monoclonal anti-OX42 (Abcam, 1:500); rabbit polyclonal anti-Iba1 (DAKO, 1:1,000); mouse monoclonal anti-CSPG (CS-56) (Sigma, 1:100), goat polyclonal anti-decorin (R&D Systems, 1:20, for immunochemistry and 5μg/ml as a neutralising antibody), rabbit polyclonal anti-decorin (Abcam, 1:50), mouse monoclonal anti-Osteopontin Invitrogen (ThermoFisher, 1:250); mouse monoclonal anti- integrin α9 (Abcam, 1:250); mouse monoclonal anti-tenascin-C (Abcam, 1:300). Secondary antibodies for immunohistochemistry were AlexaFluor™ 594 and AlexaFluor™ 488 (1:1,000, Molecular Probes, Life Technologies). DAPI was used to stain nuclei (1 μg/ml, Sigma Aldrich). Alexa 488-tagged isolectin-B4 (Molecular Probes Life Technologies, 1 mg/ml) was used to identify IB4 neurons in the DRG.

### Confocal microscopy

Multichannel fluorescence (DAPI–FITC–Texas Red filter set) images were captured using a Zeiss LSM 700 laser-scanning confocal microscope. For high-magnification images, a 63× oil-immersion Aprochromat objective (Carl Zeiss) was used. Settings for gain, aperture, contrast, and brightness were optimised initially and held constant throughout each study so that all sections were digitised under the same conditions of illumination. Channels were imaged sequentially to eliminate bleed-through, and multichannel image overlays were obtained using Adobe Photoshop 7.0 (Adobe Systems).

### ELISA S100B

The enzyme-linked immunosorbent assay (ELISAs) were carried out using the ELISA Kit for S100 Calcium Binding Protein B (S100B), Cloud-clone Corp. SEA567Ra, according to the manufacturer's instructions.

### Analysis of RARβ2 expression in human WBCs

Details of the Phase I trial are previously described (Goncalves et al., 2023). Ten millitre blood sample was taken from each volunteer according to the standard operating procedure in tubes containing anticoagulant Ethylenediaminetetraacetic acid (EDTA) and RNAlater (RNA extract) from the RNeasy mini kit (Qiagen 74104). Samples were centrifuged at ~1,500–2,000×*g* for 10–15 min at room temperature with no brake applied. Aliquots of plasma were then stored at −80°C until used. Reverse transcription was carried out in a LightCycler 480 SYBR Green I Master (Roche 04707516001), using the QuantiTech Reverse Transcription kit (Qiagen 205311). PCR reactions for the reference gene (glyceraldehyde-3-phosphate dehydrogenase; GAPDH) and RARβ2 from the same sample were run on the same plate. Each reaction was run in triplicate, and relative expression values were obtained using a GAPDH standard curve. Primers used were human GADPH forward gttcgtcatgggtgtgaacc and rev gcatggactgtggtcatgagt, product size 142 bp; human RARb2, forward tctacactgcgagtccgtct, and reverse tcaattgattgagcagtgtgc, product size 105 bp. Products were identified by melting curve analysis.

### Statistical analysis

Data and statistical analysis comply with the recommendations on experimental design and analysis in pharmacology (Curtis and Abernethy, 2015). Data analysis was performed in a blinding fashion. All statistical analysis was performed using Sigma Stat Software (SPSS Software Ltd., Birmingham, UK) using unpaired *t*-tests and one-way ANOVA with Pairwise Multiple Comparison Procedures (Tukey Test), or two-way ANOVA with Pairwise Multiple Comparison Procedures (Holm–Sidak method) as indicated in the Figure legends—^*^p ≤ 0.05, ^**^p ≤ 0.01, and ^***^p ≤ 0.001. Exact *p*-values are shown when *p* > 0.001. *n* represents the number of biological replicates. The experiments were repeated to ensure the reproducibility of the observations. No statistical methods were used to predetermine the sample size.

### Statistical analysis and processing of pharmacokinetic and pharmacodynamic parameters

The data processing and statistical analysis of the results were performed by Richmond Pharmaceuticals Ltd. in conformity with Richmond Pharmacology Standard Operating Procedures.

The PK parameters were computed using WinNonlin™ PK software (version 6.3 or higher, Pharsight Corp., Sunnyvale, California, USA).

## Results

### C286 modulates various regenerative pathways and restores levels of ECM and cell adhesion molecules in the injured SC

To achieve an unbiased, genome-wide view of transcriptional profiles between C286 and vehicle-treated spinal pathways, we performed a comparative co-expression analysis of genome-wide mRNA sequencing of SCs isolated from non-injured and avulsed male rats that had been treated with vehicle or C286 after 4 weeks of treatment with oral dosing of 3 mg/kg or vehicle every other day (Figure 1A). Non-injured tissue was used to establish normal gene expression with and without C286 treatment. Through analysis of co-expression pathways, we have identified 82 genes presenting a return to their basal level after C286 treatment of injured animals (path 4), as well as other 99 genes being strongly and specifically upregulated under these conditions (path 2). A similar situation is observed for downregulated genes, where 58 genes are returned to their basal level due to the C286 treatment after being significantly downregulated under injured conditions (path 5); and 23 genes are specifically downregulated during these conditions (path 7; Figure 1B).

Among the aforementioned co-expression paths, genes associated with the ECM were the most significantly modified, such as the extracellular region, glycoprotein extracellular matrix, proteinaceous extracellular matrix, extracellular transmembrane, collagen fibril organisation and extracellular matrix structural constituent paths (Figures 1C–F). Paths within this signalling complex are both upregulated (Figures 1C, E) and downregulated (Figures 1D, F) by the drug after injury. Amid the upregulated pathways were those pertaining to the modulation of the cholinergic system, ion transport, and ion channels (Figure 1C). Cholinergic SC neurons are essential for all aspects of motor control, including voluntary contractions of the limbs and involuntary motions of internal organs (Alkaslasi et al., 2021), and galantamine, an acetylcholinesterase inhibitor, improves functional recovery and reduces lesion size in a rat model of SCI (Sperling et al., 2019). In addition, important cholinergic modulatory loci, C boutons, express ion channels, suggesting these are important to regulate C bouton-mediated excitability during locomotor-related activity (Miles et al., 2007). Ligand-gated ion channels were also highly differently upregulated by the agonist. These constitute an important class of plasma membrane proteins recognised as critical for mediating cell-cell communication and cellular excitability since their expression on axons near presynaptic sites of release provides a direct signalling pathway to locally modulate the strength of synaptic transmission between neurons independent of the somatodendritic compartment (Engelman and MacDermott, 2004). G-protein coupled receptor signalling cascades and G-protein coupled receptor protein pathways and chemokine signalling, which are also regulated by C286, are involved in axonal pathfinding (Xiang et al., 2002; Li et al., 2005). EGF signalling inhibits axonal outgrowth, and this pathway is downregulated by C286 in the injured SC (Onesto et al., 2021).

The molecular components of the extracellular space and their interactions with transmembrane proteins are particularly important determinants of growth cone and axonal guidance, and these pathways were amongst the most significantly modulated by the agonist. Due to their potential role in influencing regeneration, we chose to further study three extracellular molecules: TN-C, integrin α9 and OPN. The main glycoproteins in the mature CNS extracellular matrix are TN-C and OPN, both of which are upregulated after injury. TN-C is upregulated after dorsal root (Pindzola et al., 1993; Golding et al., 1999) and SCI (Zhang et al., 1997; Tang et al., 2003) and can have opposing effects on regeneration depending on the presence or absence of other transmembrane cell adhesion molecules, such as integrins expressed along the axonal membrane (Bartsch, 1996; Meiners et al., 1999; Joester and Faissner, 2001; Rigato et al., 2002). Integrins are heterodimeric transmembrane receptors consisting of an α and β subunit that mediate cell adhesion, proliferation, and migration by binding to extracellular matrix proteins. They promote neurite outgrowth in embryonic (Lein et al., 1991; Neugebauer et al., 1991), postnatal (Tomaselli et al., 1993; Vogelezang et al., 2001), and adult neurons (Condic, 2001; Gardiner et al., 2005) and are involved in peripheral nerve regeneration (Vogelezang et al., 2001; Gardiner et al., 2005, 2007). However, integrins are downregulated in the adult CNS and peripheral nervous system (PNS), and the upregulation of inhibitory molecules such as chondroitin sulphate proteoglycans (CSPGs), myelin debris, and Nogo-A in the lesioned environment results in integrin inactivation, thus relinquishing axon-promoting effects (Zhou et al., 2006; Hu and Strittmatter, 2008; Tan et al., 2011). OPN is a secreted glycoprotein with pleiotropic physiological and pathological functions that influence the adhesion, proliferation, differentiation, migration, and survival of numerous cell types (Denhardt et al., 2001; Kazanecki et al., 2007; Wang and Denhardt, 2008). Several studies indicate that OPN may be an important injury-induced, SC-secreted factor that has the potential to promote motor axon regeneration (Jander et al., 2002; Ahn et al., 2004; Küry et al., 2005).

After SCI, TN-C is produced predominantly by astrocytes in the glial scar (Fawcett and Asher, 1999), and it can promote long-distance sensory axon regeneration in the presence of integrin α9 (Yokosaki et al., 1998). OPN is necessary for regeneration of peripheral sensory and motor axons (Peeters et al., 2015) and CNS axons (Bei et al., 2016).

**Figure 2:**
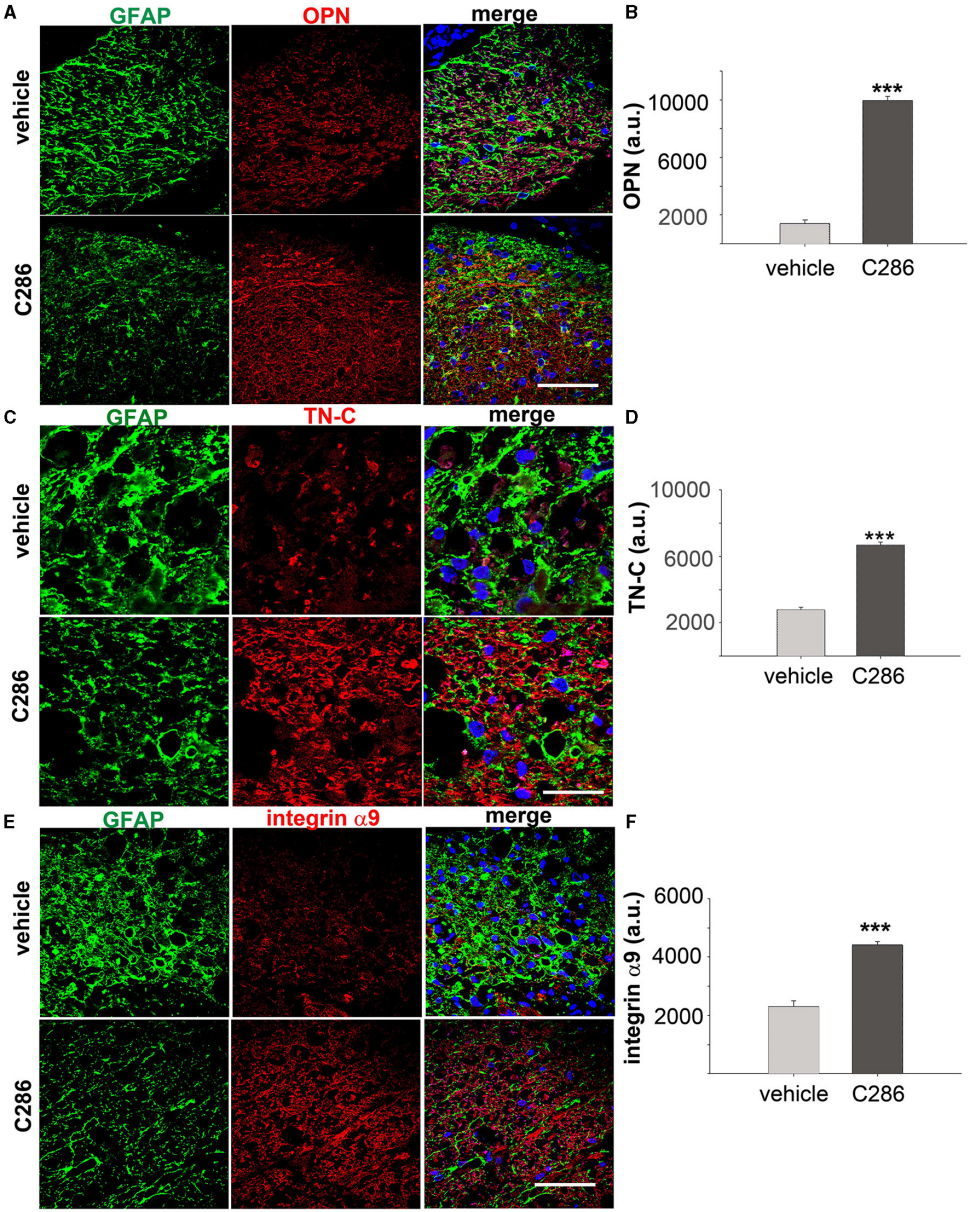
C286 upregulates Tenascin-C, alpha integrin, and osteopontin in the SC of avulsed rats. **(A–F)** Expression and quantification of TN-C, OPN and integrin α9 around the glial scar in the SC of the avulsed vehicle and C286-treated rats. Scale bars are 100 μm. Data show mean of Fluorescence Intensity (FI) ± standard error of the mean (SEM), *n* = 4 per treatment group, 5 sections per animal, Student's *t*-test, and ***p ≤ 0.001.

We found that integrin α9, TN-C and OPN were all significantly upregulated in the SC of avulsed C286-treated rats compared to vehicle-treated ones, and they were highly upregulated in and around astrocytes from the glia scar (Figures 2A–F).

### Efficacy and mechanisms of C286 in spinal contusion

**Figure 3:**
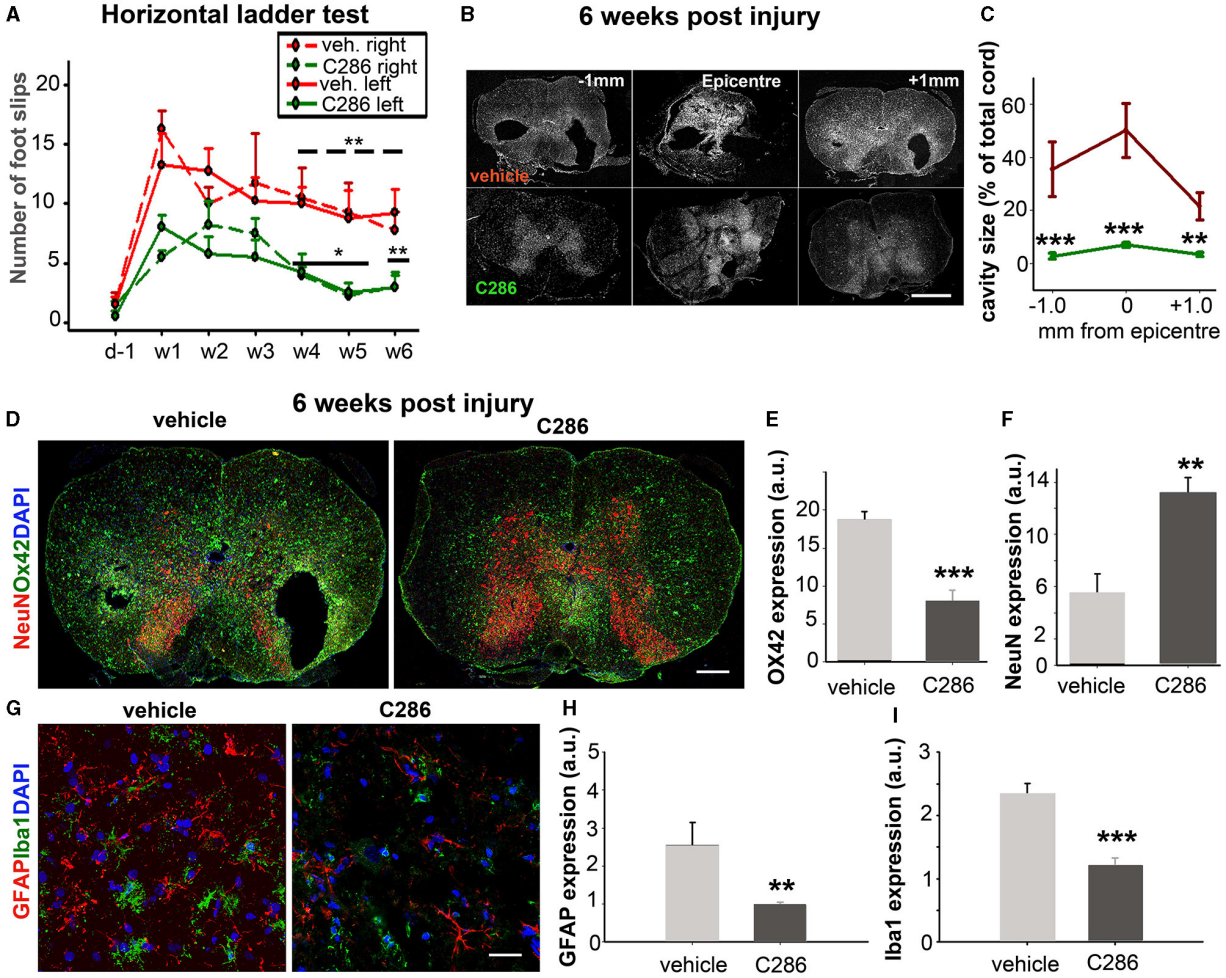
C286 induces functional recovery and tissue sparing in a model of spinal cord contusion. **(A)** C286 treatment leads to significant improvements in locomotor function in hind limbs in comparison to vehicle-treated rats. Data represent the mean ± SEM of *n* = 4, *p ≤ 0.01, **p ≤ 0.005. “____” for the statistical significance of the right hind limbs and “___” for the left. One-way Analysis of Variance (ANOVA), followed by the Fisher test. **(B)** A schematic representation of the spinal cord area used for analysis of the injury pathology and cavity size. **(C)** Quantification of cavity area (expressed as a percentage of SC area) at 0 and 1 mm caudal to the epicentre at week 6 post-injury. Data represent the mean ± SEM, **p ≤ 0.005, ***p ≤ 0.001, and Student's *t*-test. **(D–I)** C286 treatment leads to improved injury pathology and neuroprotection following a spinal contusion. Quantification of averaged **(E)** OX42, **(F)** NeuN, **(H)** GFAP, and **(I)** Iba1 expression shown as mean pixels in arbitrary units (a.u.) measured over a series of sections from 0–1 mm caudal to the epicentre revealed a significant preservation of spinal neurons and decreased reactive glia following C286 treatment compared with vehicle treatment. Data represent the mean FI ± SEM, **p ≤ 0.005, ***p ≤ 0.001, and Student's *t*-test.

We next tested the effect of C286 in another clinically relevant model of SCI, a rat with moderate thoracic SC contusion. Given that most spinal cord injury patients have a loss of motor function due to injury to the corticospinal tract (CST) using the same dosing regimen in male contused rats as for avulsion, we assessed the locomotor function on the horizontal ladder for the treatment period plus an additional 2 weeks to evaluate the “wash-out” effect. Rats showed significant improvements in hind limb locomotor function compared to the vehicle group. These were seen from week 3 until week 6 post-injury, by which point they were comparable to pre-injury outputs (Figure 3A). Analysis of the SC cavity size 6 weeks post-injury at the lesion site and 1 mm caudally (Figure 3B) showed that C286 treatment had significantly reduced the tissue loss that progressively occurs at sub-acute and chronic stages following the injury (Beattie et al., 2000) (Figure 3C). This is largely due to the inflammatory reaction that initiates at the epicentre of the lesion and subsequently spreads, resulting in tissue loss accompanied by gliosis at the secondary cavity borders (Beattie et al., 2000). When we assessed the inflammatory state and neuronal loss of the spinal cords (0–1 mm caudal to the lesion epicentre) to evaluate the effect of C286 on the secondary sub-acute pathology, we found that the agonist significantly reduced gliosis, shown by a reduction in OX42 expression and neuronal loss assessed by neuronal nuclear protein (NeuN) levels (Figures 3D–F). It also significantly lowered the expression of reactive astrocytes Glial fibrillary acidic protein (GFAP) and microglia Ionized Calcium Binding Adaptor Molecule 1 (Iba1), the latter showing the typical reactive amoeboid phenotype (Kreutzberg, 1996) in the vehicle-treated rats only (Figures 3G–I).

**Figure 4:**
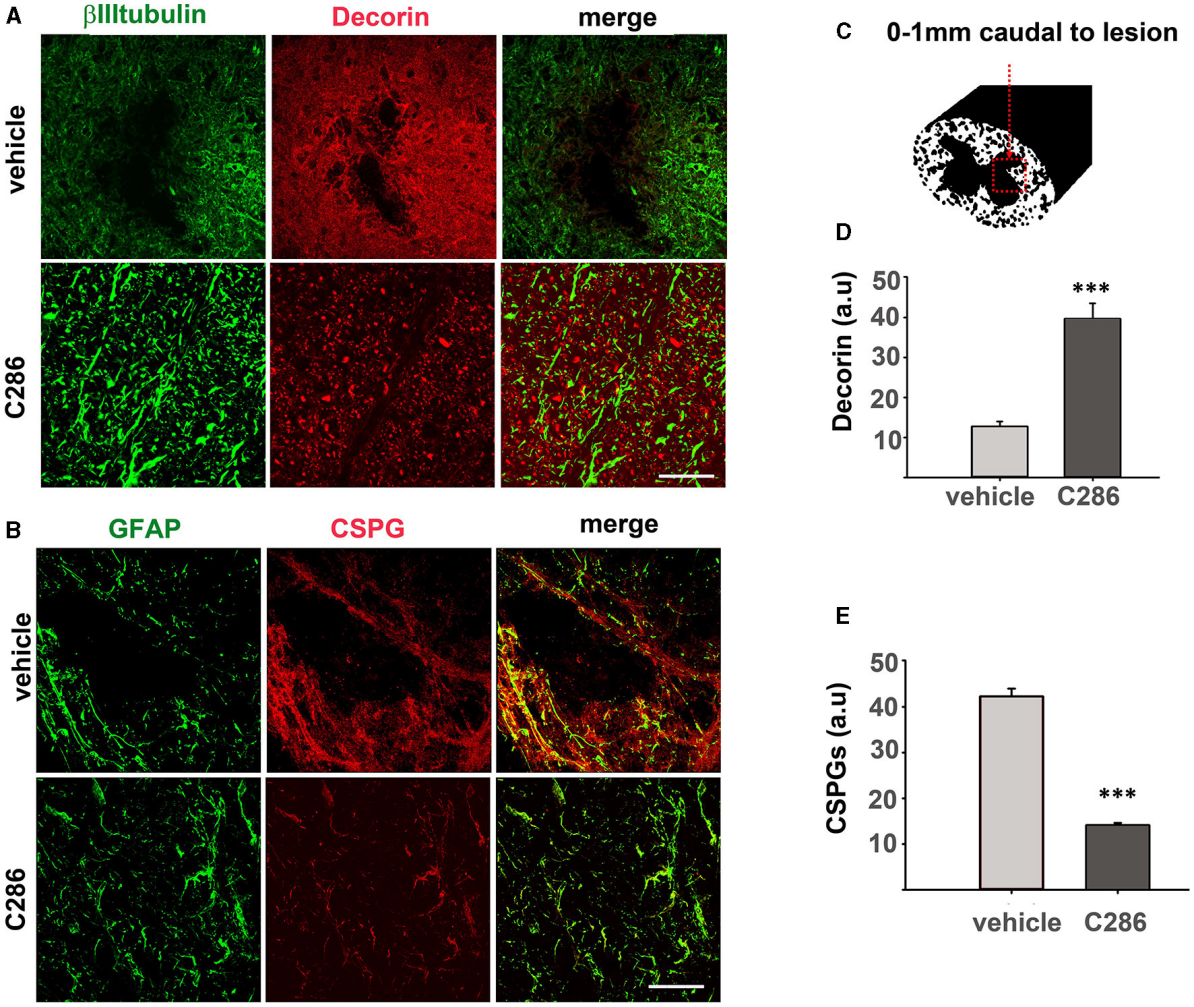
C286 remodels ECM molecules in the spinal cord after contusion. **(A)** Expression of decorin in neurons and astrocytes; **(B)** CSPGs in astrocytes; in the spinal cord **(C–E)** quantification of immunofluorescence intensity is shown as a.u. Done in a 200 μm^2^ area from sections ranging from 0–1 mm caudal to the lesion site showed that decorin was significantly higher and CSPGs significantly lower in the C286-treated rats compared to vehicle-treated ones. Results are mean FI ± SEM of *n* = 4 per treatment group, 5 sections per animal, ***p ≤ 0.001, and Student's *t*-test. Scale bars are 50 μm.

The breakdown of CSPGs using chondroitinases or decorin has been able to promote functional recovery in animal models of SCI (Esmaeili et al., 2014; Siddiqui et al., 2015). We had previously shown that C286 induces decorin in the injured SC of sensory-avulsed rats, resulting in a significant reduction in CSPGs (Goncalves et al., 2019c). To assess if this mechanism prevailed in the contused SC, we measured decorin and CSPGs as previously described (Goncalves et al., 2019c) 0-1 mm caudal to the lesion (Figure 4C). We found that decorin expression was significantly upregulated in contused rats dosed with C286 compared to vehicle-treated ones (Figures 4A, D). Concurrently, the presence of CSPGs was significantly lower in C286-treated animals (Figures 4B, E) with the RARβ agonist treatment. This suggests that C286 decreases CSPGs via an upregulation of decorin that has been shown in avulsion (Goncalves et al., 2019c).

**Figure 5:**
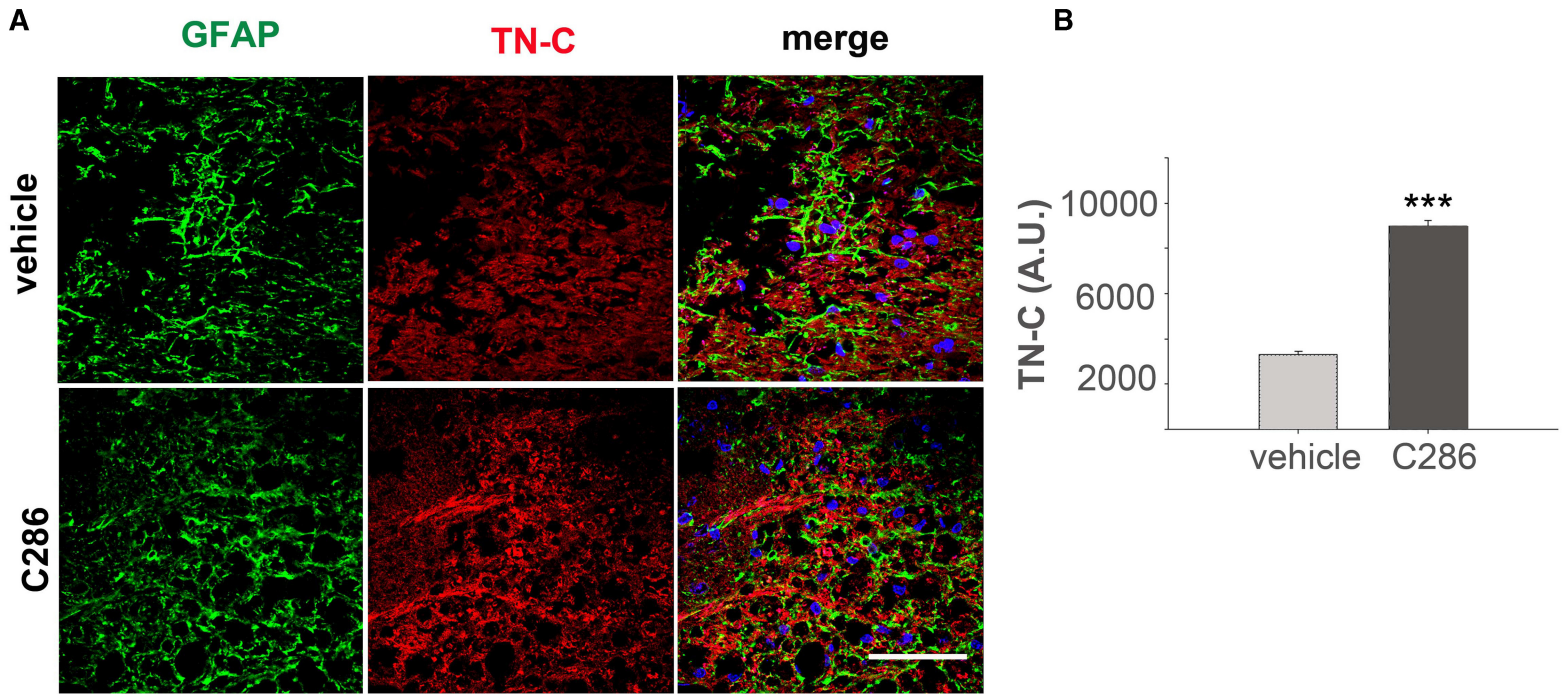
C286 upregulates Tenascin-C in the SC of spinal contused rats. **(A, B)** Expression and quantification of TN-C around the glial scar in the SC of vehicle and C286-treated spinal contused rats. Scale bar: 70 μm. Data show mean FI ± SEM, *n* = 4 per treatment group, 5 sections per animal, Student's *t*-test, and ***p ≤ 0.001.

Next, to determine if the ECM remodulation occurred in the contused cord as shown in the avulsed SC, we looked at TN-C expression in the SC of contused rats. We found that these were upregulated in the C286-treated group, suggesting the same mechanisms of action of C286 occur in different CNS lesions (Figures 5A, B).

### RARβ2 engagement by C286 in both rats and humans

**Figure 6:**
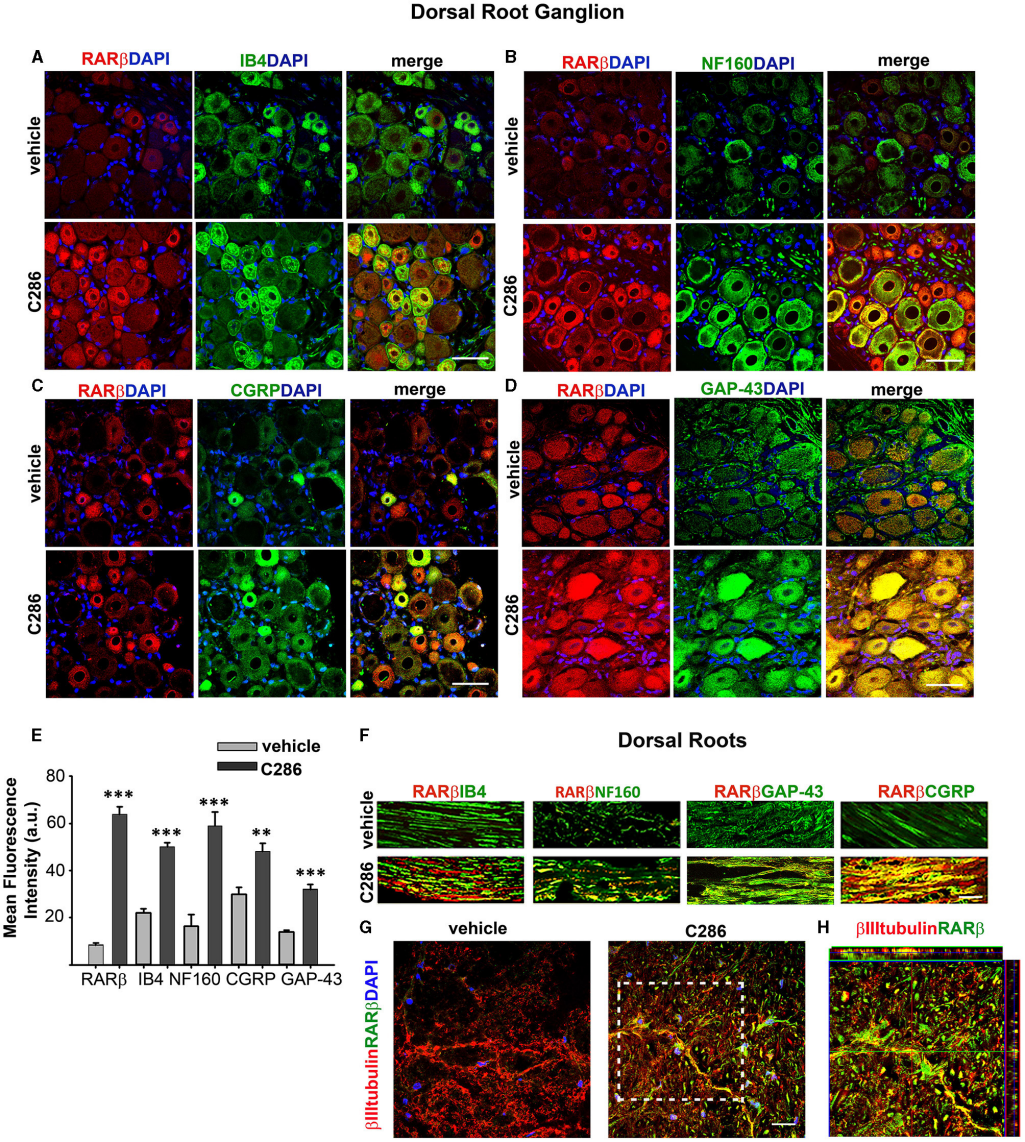
RARβ signalling stimulates the regeneration of all DRG neuron subtypes. **(A–D)** Increased co-expression of RARβ and IB4, NF160, CGRP, and GAP-43 in DRG neurons in avulsed rats compared to vehicle-treated ones. Scale bars are 50μm. **(E)** Quantification of the immunofluorescence intensities expressed as a.u. Resulting from the averages obtained from six sections per rat, taken at the same anatomical level, *n* = 4 per group. Data represent the mean FI ± SEM, **p ≤ 0.005, ***p ≤ 0.001, and Student's *t*-test. **(F)** Upregulation of RARβ, IB4, NF160, CGRP, and GAP-43 in the DRs of C286 treated compared to vehicles. **(G)** Upregulation of RARβ in neurons at the injury site. **(H)** Inset showing colocalisation of RARβ and βIII tubulin at the injury site. The scale bar is 50 μm **(F, G)**.

RARβ2 is upregulated by its agonists (Zelent et al., 1991; Kato et al., 1992). Therefore, to confirm target engagement in the avulsion model, we looked at RARβ expression in the various neuronal populations, both in the cell bodies in the dorsal root ganglion (DRG) and axons in the dorsal roots (DRs). We double-stained DRGs and DRs with RARβ and Calcitonin gene-related peptide (CGRP) to identify nociceptive small-diameter peptidergic neurons, isolectin B4 (IB4) for nonpeptidergic neurons, NF160 for large-diameter myelinated neurons (Averill et al., 1995; Snider and McMahon, 1998), and GAP-43, which is synthesised in neuron cell bodies during axonal regeneration and then transported along the regenerating axons (Benowitz et al., 1981; Skene and Willard, 1981a,b) (Figures 6A–D, F). RARβ was upregulated in the cell bodies and axons of all neuron subtypes in the agonist-treated animals compared to the vehicle-treated ones, as were CGRP, IB4, NF160 and GAP-43 (Figure 6E). In the dorsal horn axons of spinal contused rats, RARβ2 was virtually absent in the vehicle-treated rats but highly upregulated in the agonist-treated ones (Figures 6G, H), confirming target engagement at the site of injury.

**Figure 7:**
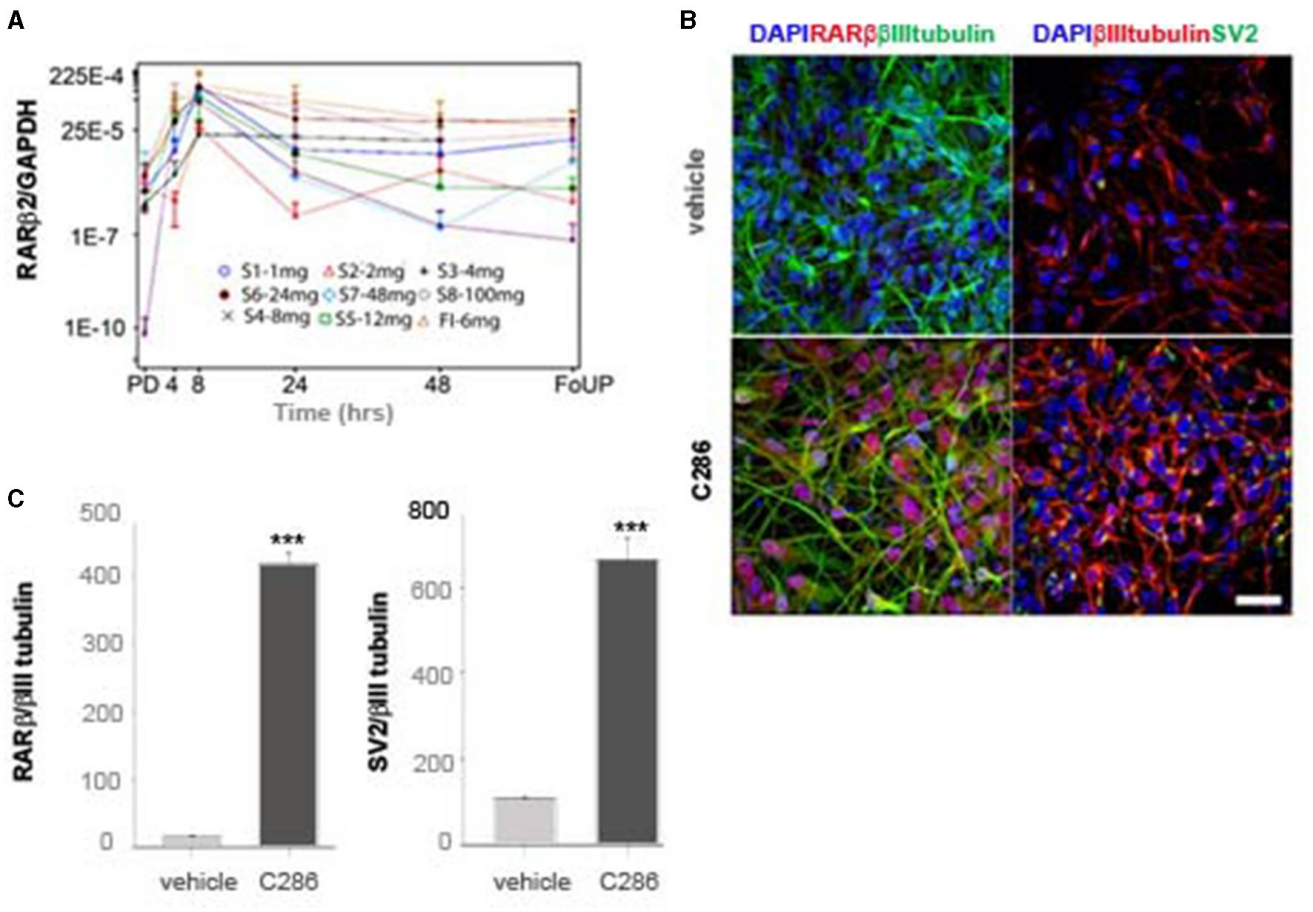
Expression of RARβ in human cells is upregulated by C286 **(A)**. **(A)** From the phase I trial of C286 administered to healthy male human participants, with increasing doses in SAD cohorts, there is an increase in the expression of RARβ2 in WBCs. **(B)** In human neurons derived from iPSCs, C286 upregulates RARβ2 expression and neurite outgrowth with increased synapse formation compared to vehicle-treated cultures. **(C)** Quantification of immunofluorescence intensity of RARβ2 and SV2 compared to tubulin expression. Error bars denote standard deviation (SD) in **(A)** and SEM in **(C)**. Ratio values equal to zero are set to missing. ***p ≤ 0.001 and Student's *t*-test. Scale bar: 50 μm. Pre-dose (PD), follow-up (FoUP).

We next determined the pharmacokinetics of C286 in the rat avulsion model. Injured rats were treated orally with C286. Exposure in terms of *C*_max_ and AUC_0 − t_ (area under the curve) was dose-related on Day 1 and Day 25 of dosing. No C286 was measurable in vehicle-dosed control animals sampled at Tmax (2 h). At the 3 mg/kg dose, sensory and motor behavioural benefits were associated with exposure to *C*_max_ 1,750 ng/ml and AUC_0 − 24*h*_ of 10,808 ng.h/ml on day 1. This was equivalent to a 100 mg daily oral dose in healthy human males (Goncalves et al., 2023) and is below the level of no adverse event level (NOAEL) in the 28-day toxicology study of 10 mg PO every day in dogs exposure to *C*_max_ 3,700 ng/ml and AUC_0 − 24*h*_ of 25,110 ng.h/ml (Goncalves et al., 2023). In the Phase I trial in the single ascending dose (SAD) cohorts, we measured the expression of RARβ2 levels in WBCs as a target engagement biomarker in male healthy participants (Goncalves et al., 2023). Within the doses administered in the study (up to 100 mg), little or no RARβ could be detected at pre-dose levels in any of the SAD cohorts (Figure 7A). There was a correlation between the dose of C286 and RARβ2 expression in WBCs, which peaked at 8 h post-dosing, with the peak being higher with increasing dose (Figure 7A). To ask if food interfered with the activation of the receptor, one cohort (FI) was fed before the administration of the 6 mg dose (Figure 7A). There was no difference in the time of activation of the RARβ receptor compared with the fasted cohorts, suggesting that the drug was as well absorbed (Figure 7A). To show that activation of the human RARβ2 by C286 can elicit a neurite outgrowth response in humans, we derived human neurons from human-induced pluripotent stem cells (iPSCs). These were cultured in the presence of 0.1 μM C286, and the density of RARβ2/βIIItubulin and amount of synaptogenesis using synaptic vesicle protein 2 (SV2) were measured 3 days after treatment. In drug-treated cultures, RARβ2 was induced by an increase in neurite density and an increase in the number of synapses to a significant extent compared to vehicle-treated cultures (Figures 7B, C).

### Plasma S100B as a putative regeneration biomarker

**Figure 8:**
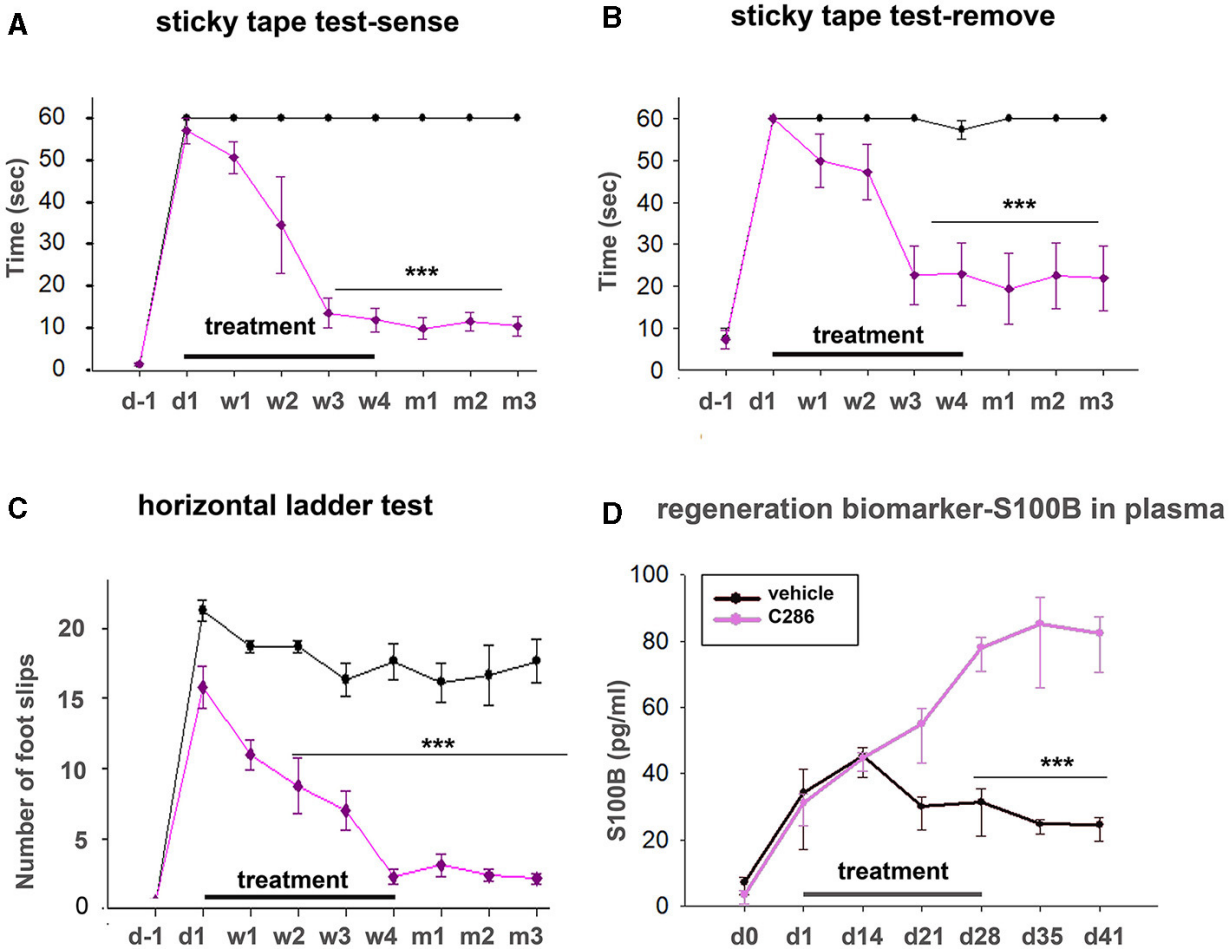
Plasma S100B as a regeneration biomarker. Locomotor and sensory recovery of avulsed rats treated with vehicle or C286 was tested for 3 months post-injury, and plasma s100b was measured up to 41 days post-injury. **(A, B)** In a tape removal task, the time taken to sense and remove the tape by the lesioned forelimbs was measured, and significantly lower latencies were observed with the injured forelimbs of RARβ agonist-treated rats compared with vehicle-treated ones from week 3 of treatment onwards. **(C)** In locomotor tasks, the number of foot slips in a horizontal ladder made by the injured forelimb of RARβ-agonist-treated rats was markedly lower than that of vehicle-treated rats from week 2 onwards. Data represent the mean ± SEM. ***p ≤ 0.001, one-way ANOVA followed by Tukey's *post-hoc* test. **(D)** S100B levels were quantified from plasma samples obtained weekly for 6 weeks. A significant increase was observed in C286-treated rats from week 3 onwards. Data represent the mean ± SEM S100B plasma concentration (pg/ml), *n* = 6–9 per treatment group, and time point. One-way ANOVA, Multiple Comparison Procedures (Holm–Sidak method), and ***p ≤ 0.001.

S100B is a member of a family of Ca^2+^-binding proteins of the EF-hand type that acts as an intracellular regulator and as an extracellular signal to exert a plethora of functional roles such as tissue development, repair, and regeneration (Sorci et al., 2013). To assess if plasma S100B levels could be an indicator of regeneration after SCI and correlate with normal locomotor and sensory activity, we treated male avulsed rats (C5-T1 dorsal root avulsion) with either vehicle or C286 (3 mg/kg, orally every other day) for 4 weeks, carried out behavioural tests for 3 months after the lesion, and measured plasma S100B until day 41 after injury. We found that full locomotor and sensory recovery had been achieved after 3 weeks of C286 treatment, and the rats remained functional thereafter (Figures 8A–C). The vehicle-treated rats did not regain function within that time window. The plasma S100B levels were significantly higher in C286-treated rats compared to vehicles from 3 weeks onwards and remained higher and stable from day 28 up to the last measurement timepoint, day 41, thus correlating with the regain and maintenance of function (Figure 8D). This suggests that S100B may be necessary for normal locomotor and sensory functions and that C286 induces its extracellular release.

### Necessity of RARβ drug for neural regeneration

**Figure 9:**
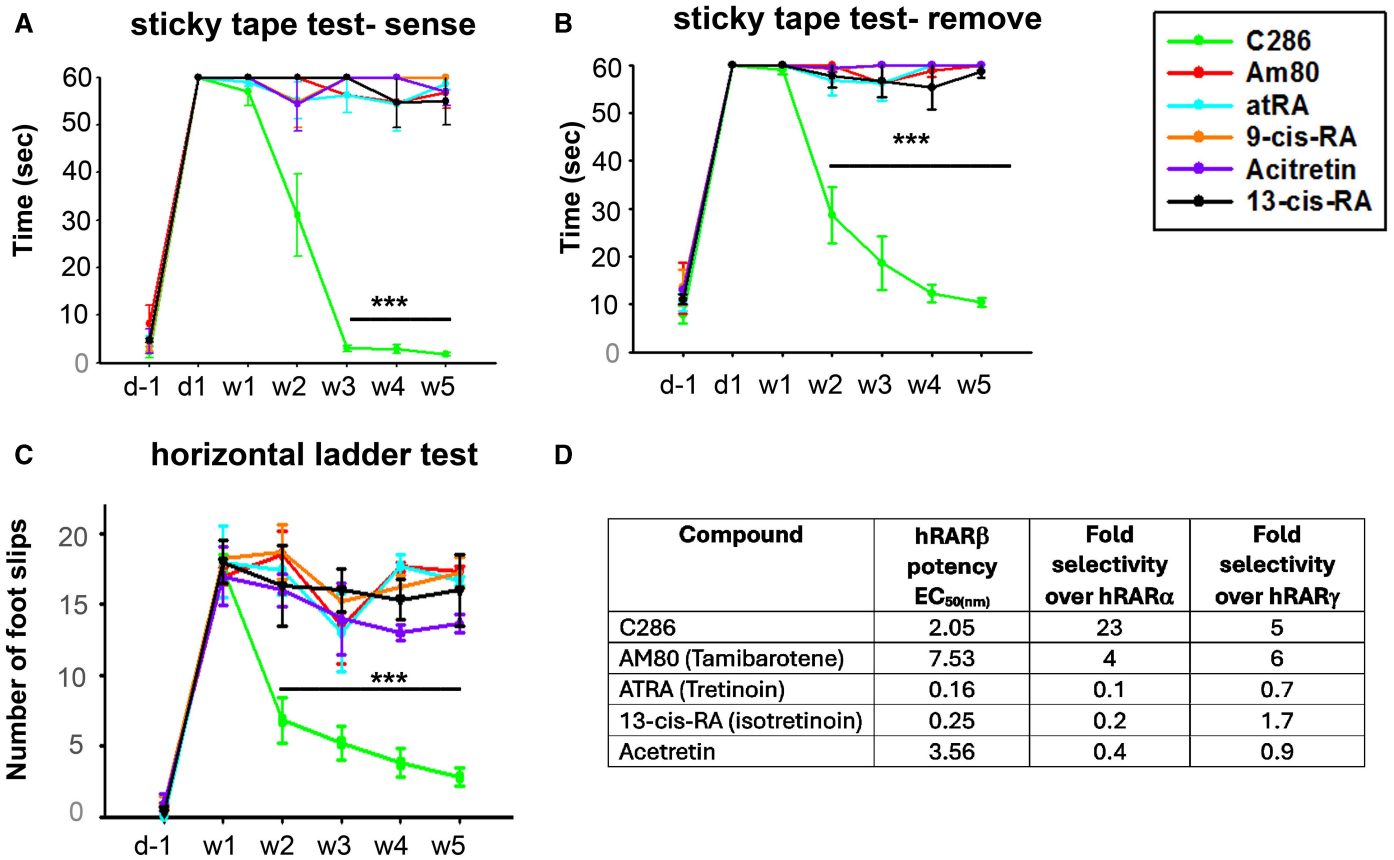
Comparison of functional recovery induced by C286 and marketed retinoids in an avulsion model and their activity profile at human RARs. Rats with a C5–C8 + T1 avulsion were treated with vehicle, C286 and commercially available retinoids (Am80, atRA, 9-cis-RA, Acitretin and 13-cis-RA) at 1 mg/kg, i.p., 3 days a week for 4 weeks. Rats were trained for 2 weeks before surgery in behavioural tasks, and scores were recorded the day before surgery, the day after surgery, and then weekly for 5 weeks **(A–C)**. Only C286 was capable of inducing significant sensory and locomotor recovery, with further improvement still being observed 1 week after cessation of treatment. Data represent the mean ± SEM of *n* = 3–6, ***p ≤ 0.001. One-way ANOVA followed by Tukey's *post-hoc* test. **(D)** Activity profile of C286 and commercially available retinoids at the human (h) RARs in an *in vitro* transactivation assay.

To validate the requirement for a specific RARβ drug for axonal regeneration, we compared the functional recovery of avulsed male rats treated with C286 and five clinically available retinoids that are not RARβ selective. Three are pan-RARs (tretinoin, isotretinoin, and acitretin), one is a RARβ/α agonist (tamibarotene) and another is a retinoid X receptor agonist (bexarotene). These retinoids have varied clinical applications, the best-known being acne treatment, either by topical application (isotretinoin) or orally (tretinoin and acitretin) (Khalil et al., 2017; Latter et al., 2019). Tamibarotene has been used for the treatment of acute promyelocytic leukaemia (Shinjo et al., 2000), acitretin for psoriasis (Pilkington and Brogden, 1992), and bexarotene for cutaneous T-cell lymphoma (Schadt, 2013). However, none have been used clinically to treat SCI. Only C286 could restore functional outputs compared to the other retinoids (Figures 9A–D), confirming the efficacy of a specific RARβ agonist to treat neural injuries.

## Discussion

Nerve injuries are a complex clinical condition for which the current treatment options have limited success in neurological and functional recovery. Significant functional recovery can only be attained by multitarget therapies that promote axonal regrowth and overcome the blocks associated with glial scarring, which represent a mechanical barrier to regrowth and the presence of inhibitory molecules (Afshari et al., 2009). Here we demonstrate that a multifactorial novel orally available Phase 2a-ready drug, C286, can induce functional recovery in rats with either sensory rhizotomised or contused thoracic SCs through alteration of gene expression patterns and reassembly of extracellular matrix molecules to harness tissue repair and axonal regeneration. C286 engages with the target in humans and could therefore be a possibly successful therapy for nerve injuries.

Neuronal RARβ signalling has long been identified as a pivotal target for axonal regeneration in various nerve injuries, including optic nerve regeneration (Koriyama et al., 2013), diabetic neuropathy (Hernández-Pedro et al., 2014), SCI (Agudo et al., 2010), and avulsion (Goncalves et al., 2015, 2019a,c). Unlike the vast majority of targets explored for SCI therapy, the neuronal RARβ signalling pathway can concomitantly change various aspects of the molecular and cellular pathology that arises after nerve injury as it modulates transcriptional patterns and has additional non-transcriptional effects. We had previously shown that RARβ activation after nerve injury suppresses neuronal Phosphatase and tensin homolog (PTEN) and astrogliosis (Goncalves et al., 2015), induces remyelination (Goncalves et al., 2019c), promotes DNA repair (Goncalves et al., 2019b), prevents the onset of neuropathic pain (Goncalves et al., 2019b), a common secondary sequelae of spinal injuries, and modulates mitochondrial function to harness axonal growth (Trigo et al., 2019). Here we show an additional set of pathways that are modified by C286; these include axonal guidance, modification of the ECM, and the formation of synapses, which are necessary to regenerate functioning neurons (Curcio and Bradke, 2018; Zheng and Tuszynski, 2023).

Whilst our previous study demonstrated the efficacy and explored some of the mechanisms by which specific activation of neuronal RARβ induces functional locomotor and sensory axonal recovery in a rat model that mimics brachial plexus avulsion (BPA), which is the most severe form of axotomy, here we extend our study to spinal cord contusion. The prevalence rate of BPAs is ~1.2% of multiple traumatic injuries, whereas the global prevalence of SCI is considerably higher, between 236 and 1,009 per million (Yang et al., 2014; Peeters et al., 2015).

Here we show for the first time a preclinical POC study for C286 in spinal contusion and demonstrate its effects on important aspects of spinal trauma secondary neuroinflammation and in the remodulation of extracellular matrix molecules. This multifactorial action should not be surprising, as retinoids are well-known as transcriptional agents with access to a substantial part of the genome (Luo et al., 2009), and the genes that neuronal RARβ regulates must be the same irrespective of the type of nerve injury resulting in the same pathways being regulated for benefit.

Preventing or diminishing secondary neuroinflammation after spinal trauma not only limits tissue damage but also creates a molecular milieu that is more conducive to axonal regrowth. Equally, the post-trauma ECM environment is a recognised barrier to axonal regeneration in the adult nervous system. TN-C can provide an axonal permissive structural infrastructure as long as it is bound to the appropriate TN-C binding molecules, such as integrins (Yokosaki et al., 1998). These are downregulated after development, and other inhibitory molecules that populate the lesioned area, such as CSPGs and Nogo-A, inactivate them (Zhou et al., 2006; Hu and Strittmatter, 2008; Tan et al., 2011). Here we show that C286 remodels the post-trauma ECM environment through two converging but distinct mechanisms. It scavenges CSPGs by upregulating of decorin, a mechanism previously identified in sensory root avulsion (Goncalves et al., 2019c), and it induces the concomitant upregulation of TN-C, integrin α9 and osteopontin (OSP) in the injured SC, thus creating the necessary conditions for axonal growth through the glial scar. One of the mechanisms of OSP has been shown to involve preventing inflammation and allowing axonal outgrowth to occur (Hashimoto et al., 2007), and it does this by interacting with integrins (Zhou et al., 2020). It is not known if the upregulation of these pathways is indirectly or directly regulated by C286.

We have further shown here that C286 prevents inflammation by downregulating GFAP and Iba1, and as expected given that C286 is a genomic drug, similar pathways have been identified in the neuropathic pain model (Goncalves et al., 2019b). However, other inflammatory molecules are expressed, such as chemokines that are regulated by C286 as shown in our pathway analysis, and it will be of interest to correlate these with the axonal regeneration process.

We have not identified any detrimental pathways that the drug regulates, and this is in keeping with our 28-day toxicology data, where no neuronal deficits were identified in either a dog or rat below the level of NOAEL (Goncalves et al., 2023) or were any neurological adverse events (AEs) related to the drug dose identified in healthy participants in the Phase I trial (Goncalves et al., 2023). The fact that specific activation of RARβ is necessary, as other clinically available retinoids, not RARβ-selective, failed to improve functional recovery in lesioned rats, excludes these currently available drugs as potential candidates for the treatment of nerve injuries. This does not preclude the importance of other RARs in aspects of neural repair. For example, RARα is necessary for myelination (Goncalves et al., 2019c), but it is only when it is activated in oligodendrocytes after neuronal RARβ signalling that the coordination of axonal outgrowth and myelination can occur, suggesting that RARβ and RARα signalling act in a cell- and time-specific manner in which RARβ is hierarchically upstream of RARα concerning neural regeneration.

With respect to therapeutic translatability, the retinoid system is highly conserved between species, and RARβ has been demonstrated to preserve function across species (Escriva et al., 2006). The role of RARβ in neurite/axonal outgrowth is evolutionarily conserved, being present in rodents, avians and amphibians (Plum and Clagett-Dame, 1996; Corcoran et al., 2000; Yip et al., 2006; Carter et al., 2011; Puttagunta et al., 2011) and we have shown here its role in human neurite outgrowth. Adult nerve regeneration may simply be a recapitulation of the development of the nervous system, as there is a wave of RARβ expression that coincides with neurogenesis in the developing rodent that ends once the process is complete (Yamagata et al., 1994). However, unlike the regenerating system, high levels of retinoids must be present for developmental axonal outgrowth to occur. It is also worth noting that in some neurodegenerative diseases, RARβ signalling has been shown to have therapeutic benefits in rodent models of these diseases (Lee et al., 2014; Medina et al., 2020; Ciancia et al., 2022). Loss of axons and synapses can be restored, suggesting the same RARβ pathways may be involved as in acute SCI.

Target engagement was confirmed in rats with lesioned neurons and in healthy human participants who received the drug at doses ranging from 1–100 mg. Target engagement in humans was measured peripherally by quantification of RARβ in WBCs (Goncalves et al., 2023), and it is correlated with the dose administered. The maximum dose in the SAD, 100 mg, gives the highest level of expression of RARβ2 8 h after dosing, which then declines with time. This dose is equivalent to AUC and *C*_max_ in the rat POC studies in avulsion and contusion to give a behavioural benefit. Since C286 has a blood–brain penetration ratio of 1:1 (Borthwick et al., 2020), a pharmacological effect in patients is expected. For behavioural recovery in nerve-injured rats, the *C*_max_, and AUC_0 − 24*h*_ are equivalent to 1.5 mg/kg in humans (Goncalves et al., 2023), and these Pharmacokinetics (PK) values are twofold below the NOAEL in dogs (Goncalves et al., 2023), suggesting that the therapeutic index is 2, which is a good safety margin for subsequent Phase IIA trial.

We suggest that only a 4-week treatment with the drug is necessary to initiate an axonal outgrowth response, as we have previously shown that once the neuronal RARβ is activated, a feedback loop is established between the glia, which synthesises and secretes RA, and the regenerating neurons to keep neuronal RARβ activated until regeneration is complete (Goncalves et al., 2019c). We have also shown here that once regeneration occurs, there is no need to maintain drug dosing to maintain the regenerative response, as up to 3 months after the final dose of the drug, there is no deterioration in functional behaviour. However, these predictions can only be confirmed in humans during a Phase IIA trial.

S100B is the most widely investigated biomarker in the assessment of traumatic brain injury (Pandor et al., 2011). In the CNS, S100B is predominantly expressed in astrocytes, and after traumatic brain injury (TBI), it is released and/or leaked from the CNS to the peripheral bloodstream. S100B is also expressed in Schwann cells in uninjured peripheral nerves and activated Schwann cells up to day 7 post-injury. Non-activated Schwann cells later expressed S100B during the regeneration period in the lesioned, proximal and distal areas (Spreca et al., 1989). Increasing evidence suggests that at the concentration found in extracellular fluids in normal physiological conditions and locally upon acute tissue injury (≤50 nmol/L S100B) (Van Eldik and Wainwright, 2003; Donato et al., 2009, 2013), S100B exerts trophic effects in the central and peripheral nervous systems and reduces microglia reactivity (Sorci et al., 2013). We show here that C286 induces extracellular release of S100B and that within certain plasma concentrations (~50–90 pg/ml), S100B correlates not only with axonal regeneration and functional recovery but also with the maintenance of normal locomotor and sensory functions. In contrast, in TBI levels of S100B correlate with mild injury; this is thought to be due to inflammation, and it may be that once the levels of S100B are too high, this is detrimental to repair (Pandor et al., 2011). It may be that C286 can both upregulate and downregulate S100B to maintain optimal concentrations for neuronal repair.

In summary, we have demonstrated here that an orally available RARβ agonist drug overcomes the major hurdles foiling SCI therapeutic development: a suitable drug delivery route and a single target with multifactorial effects desired as therapeutic aims in a variety of nerve injuries. This, together with target engagement in humans, places C286 as an attractive drug to be further tested in POC trials for SCIs and other nerve injuries such as TBI, stroke, and multiple sclerosis (MS).

## Study limitations

We have defined an efficacy dose for motor and sensory recovery and identified a set of pathways associated with C286 treatment in rodent models of SCI. However, our analysis corresponds to a transcriptional and protein expression snapshot of the SC after 4 weeks of treatment; thus, it is impossible to assess the fluidity of transcriptional and molecular changes at earlier time points. This could provide insights into the chronology of the sequential pathways that regulate regeneration and could lead to the identification of novel therapeutic targets that were missed with our study design. In addition, the rat model studies were all in males. The pathways may differ for female species.

## Data Availability

The datasets generated during and/or analyzed during the current study are available from the corresponding author upon reasonable request. Raw and processed transcriptome datasets are available under the Gene expression omnibus (GEO) accession number GSE268237, https://www.ncbi.nlm.nih.gov/geo/query/acc.cgi?acc=GSE268237.
